# Rheological, Aging, and Microstructural Properties of Polycarbonate and Polytetrafluoroethylene Modified Bitumen

**DOI:** 10.3390/polym14163283

**Published:** 2022-08-12

**Authors:** Muhammad Ansar, Muhammad Ali Sikandar, Fadi Althoey, Muhammad Atiq Ur Rehman Tariq, Saleh H. Alyami, Samah Elsayed Elkhatib

**Affiliations:** 1 Department of Civil Engineering, CECOS University of IT and Emerging Sciences, Peshawar 25000, Pakistan; 2 Department of Civil Engineering, Najran University, Najran 66454, Saudi Arabia; 3 College of Engineering and Science, Victoria University, Melbourne, VIC 8001, Australia; 4 Institute for Sustainable Industries & Liveable Cities, Victoria University, Melbourne, VIC 8001, Australia; 5 Mechanical Engineering Department, Faculty of Engineering and Technology, Future University in Egypt, New Cairo 11835, Egypt

**Keywords:** BBR, bitumen aging, DSR, rheology, rotational viscosity

## Abstract

Deterioration of asphalt pavements due to massive load of vehicles and climatic variation has demanded the use of pavements construction material with an excellent resilience characteristic, resistance to permanent deformation, and most importantly, a much longer service lifespan. The main structural distresses in pavement construction are permanent deformation at high temperatures and fatigue cracking under repetitive traffic loadings. To comprehensively investigate the performance of bitumen penetration grade (PG) 70 against rutting, fatigue, and high temperature cracking in hot mix asphalt (HMA) pavements, polycarbonate (PC) and polytetrafluoroethylene (PTFE) were used. The investigation of the internal structure, rheological, and physical properties of base and modified bitumen (MB) mixes with different percentages of modifiers (0%, 2.5%, and 5%) by weight were performed via scanning electron microscope (SEM), Fourier transform infrared spectroscopy (FTIR) analysis, X-ray diffraction (XRD) pattern analysis, rolling thin-film oven test (RTFOT), pressurized aging vessel (PAV), dynamic shear rheometer (DSR), rotational viscosity (RV), and bending beam rheometer (BBR). The results of the RV test indicate that modification of neat bitumen with polycarbonate and polytetrafluoroethylene increased the viscosity for polycarbonate-modified bitumen (PCMB), polytetrafluoroethylene-modified bitumen (PTFEMB), and for a blend of PCMB-PTFEMB by 44%, 50%, and 55.75% at 135 °C and 111.10%, 127.80%, and 138.88% at 165 °C, accordingly. BBR test results revealed that modifiers increased the rigidity of neat bitumen by 74.8%, 75.8%, and 74.5% at −16 °C, −22 °C, and −28 °C, respectively.

## 1. Introduction

Bitumen has been traditionally used as a base binding material in the construction of road pavement and maintenance for thousands of years to provide sufficient workability, coating, and binding of aggregates, generally owing to its exceptional adhesion to aggregates [1,2,3,4,5]. Depending on various climates, traffic flow profiles, or design approaches, both thermal and physical parameters of the pavements should fulfill the needs of the common user. The massive increase in traffic density worldwide over the last few decades has led to an increase in premature pavement failures. Catastrophic distortion at hot temperatures, fatigue fracturing at intermediate temperature levels, and thermal degradation at relatively low temperatures are the three most common types of pavement failure [6,7,8,9,10,11,12]. Early rutting failures of pavements are one of the most serious pavement distresses, primarily due to high ambient temperatures and uncontrolled axle load. The main reasons for cracks occurring in the flexible pavement are the stiffening and embrittlement of the bitumen due to aging with the service life of pavement [13,14,15,16,17,18].

In recent decades, much emphasis has been put on the construction of composite bitumen-polymer materials in addition to enhancing the characteristics of traditional bitumen by delivering improved properties for structural and functional applications such as aging resilience, thermal sensitivity, ductility, and dimensional stability [18,19,20,21,22]. Pavement engineers can effectively comprehend the extensive mechanical performance of bitumen if they have a comprehensive and detailed knowledge of its micro-mechanical nature, especially micro-friction, micro-abrasion, and phase separation. Several researchers have reported that the nature of bitumen’s microstructure directly impacts its surface morphology and micromechanical efficiency. Bazlamit et al. used numerous experimental research to examine variations in bitumen pavement friction, while Fischer et al. utilized Scanning Near-Field Optical Microscopy, SNFOM, to evaluate the interaction between chemical compositions and micro-mechanical performance of asphalt mixture [23,24]. A micro-damage repairing model was also suggested, which offers a more reliable estimate of fatigue life in the bituminous mixture [25]. It is determined from their findings that the macro-mechanical efficiency may be influenced by the micro-mechanical nature of bitumen and the chemical content of the bitumen blend. Premature failures of flexible pavement are responsible for reducing the lifespan of the pavements and increasing the maintenance costs.

To sustain asphalt pavement with longer service life, greater rutting resistance and thermal degradation, reduction in residual stresses, stripping and temperature vulnerability, and lower maintenance activities, it is important to fully characterize or understand the rheological micro-structural properties of asphalt binders with modifiers [26]. In other contexts, the analysis of the identification of microstructural characteristics is necessary in order to strengthen the understanding of the macroscopic behaviors of the mix. The latest research reveals that the utilization of thermoplastic-based admixtures in bitumen modification has grown substantially. In a recent study, two notable groups of polymers, elastomers, and plastomers have been extensively used. Elastomers were used to raise the relatively low temperatures cracks and fatigue sensitivity of the base asphalt, while plastomers have typically been used to maximize permanent deforming resistance and enhance the elastic characteristics of the binders. In particular, rutting and aging resistance can be increased with commonly used modifiers, including styrene-butadiene rubber (SBR) and styrenebutadiene-styrene copolymers (SBS) [27,28,29,30]. Importantly, the poor compatibility of bitumen with modifiers affects pavement construction quality and thus will affect the performance of pavement. Additionally, to overcome these issues, it is essential to acquire a certain exceptional advancement. An effort has been made in this study by using two different thermoplastic synthetic polymers, namely “Polycarbonate and Polytetrafluoroethylene”, as bitumen additives to modify asphalt binders and to determine their effects on adhesive rheology and microstructural properties of polymer-modified bitumen. PC is among the most widely used engineering thermoplastic polymers due to its transparency, exceptional resilience, thermal resistance, and high dimensional uniformity [31,32]. Polycarbonate is typically manufactured from Bisphenol A (BPA; C_15_H_16_O_2_) and Phosgene (COCl_2_), using a step-growth polymerization process that excludes Cl-ions each time the monomers react. PTFE is a synthesized thermoplastic polymer (STP) that is mostly used as a coating due to its unique combination of chemical and mechanical properties, including low-temperature flexibility, low friction coefficient, high-temperature stability, strong chemical resistance to corrosive reagents, chemical reactivity in most organic solvents, long-term environment conditions, no flammability, and hydrophobicity [33]. PC and PTFE are polymers that comprise functional groups and may shape chemical bonds with many other bitumen components. Interaction between polymers and pure bitumen produces a chemical reaction with pavement binder and develops a network surrounding asphaltenes in a mixture of bitumen to create an integrated compound [34,35,36,37,38,39]. It has been investigated that the use of thermoplastic PC and PTFE-polymers for modification of neat asphalt increased the softening point temperature (SPT) values, flash and fire point temperature ranges, and viscosity of base bitumen, while decreasing its penetration and ductility [40,41,42,43].

The prime goal of this research was to examine how PTFE and PC polymers effects the physical, rheological, chemical, and microstructural properties of grade 70 bitumen with all its characteristics. In addition, to determine the rheological, mechanical, and physical characteristics of polymer-modified bitumens (PMBs), tests such as penetration, SPT value, flash point temperature (FPT1), and fire point temperature (FPT2), ductility, rotational viscosity, bending beam rheometer, and DSR test were performed. A comprehensive study was performed to understand the rheological, mechanical, and physical characteristics, microstructural behavior, and chemical composition of PMBs, using penetration, ductility, rotational viscosity, BBR, DSR, XRD, SEM, and FTIR spectroscopy.

## 2. Materials and Methods

### 2.1. Material

#### 2.1.1. Bitumen and Modifiers Selection

Many studies have been undertaken to improve the effectiveness of HMA by modifying the bituminous material by utilizing several modifiers. In this study, the base material of PG 70 was modified using two well-known thermoplastic polymers, PC and PTFE. Both mechanical and physical characteristics of neat bitumen, obtained from Attock Refinery Pakistan (Attock, Pakistan), PC and PTFE powder, supplied from Zhenjiang Co., Ltd. (Jiangsu, China), presented in Table 1, Table 2 and Table 3. PC is a high-performance thermoplastic amorphous polymer containing carbonate groups in their chemical structures. It shows good chemical and physical properties, such as excellent transparency, high impact resistance value, ductility, and chemical inertness at low and moderate temperatures.

PTFE is a synthesized thermoplastic polymer belonging to the fluoropolymer of tetrafluoroethylene plastics group. Because of its low friction coefficient and cost-effective manufacture, PTFE is utilized as an anti-stick covering for pans and other cookware. Its lightness, excellent thermal reliability, and corrosion resistance are numerous reasons for its industrial application [32].

#### 2.1.2. Preparation of PTFE and PC-Modified Bitumen

In order to produce a homogeneous asphalt blend, it is important to set up an extensive, long-term, and low-cost approach. For the preparation of polymer-modified bitumens in this study, the melting approach was followed. Three separate amounts (0%, 2.5%, 5% by weight) of bituminous additives, PC and PTFE, were deliberately chosen, considering the maximum amounts suggested by the producer to modify bitumen. All the PMBs samples were prepared by mixing in a mixer at a temperature of 180 °C for approximately 60 min at a mixing rate of 1000 rpm. After preparing PMBs samples, micro-structural and neat bitumen experiments were performed to evaluate the engineering characteristics of the PMBs samples with three different percentages. Testing specimens of Control (C), (PCMB), (PTFEMB), and (PCMB-PTFEMB) were adequately prepared and identified as C, 2.5PC, 5PC, 2.5PTFE, 5PTFE, and 2.5PC-2.5PTFE accordingly.

### 2.2. Testing Methods

#### Rheological, Mechanical, and Physical Properties

##### Conventional Tests Methods of PMBs

In this research, to examine the influence of modifiers type, PC and PTFE, and the concentration of the modifiers (0%, 2.5%, 5%) on the physical parameters and rheology of the traditional pavement binder and polymer-modified bitumen samples, various laboratory experiments, namely SPT value test, flash and fire value test, PG (25 °C) value test, and ductility (15 °C) were conducted in line with the ASTM methods D36, D92, D5, and D113, respectively. Moreover, the results of SPT and PG value tests were performed to calculate the penetration index (PI) value [44]. When utilizing the bituminous material with a higher penetration index value, the asphalt blend reveals more resistance to deformations and thermal cracking [45].

##### Short-Term Aging (STA) and Long-Term Aging (LTA) Methods of Binders

The process of aging of bituminous samples occurs at various stages, including during processing, mixing, and over the pavement’s operating period. The bitumen oxidizes when mixed at higher shear levels or for longer periods. The bitumen aging process is separated into two parts: the first one is associated with bitumen volatilization as well as the intensity of aging that occurs in bitumen binder throughout mixing and construction stages, and therefore is described as short-term aging; the second is described as long-term aging of binders in which slow oxidizing of the material occurs beyond a period of 8–10 years following the construction of the pavement structure. In this study, RTFOT and PAV experiments were conducted to determine the short-term and long-term aging of bitumen samples accordingly in the laboratory.

##### Dynamic Shear Rheometer Test

The complex modulus (G*) and phase angle (δ) of the thermoplastic polymers, PC and PTFE-modified bitumen, and base binder were calculated using the DSR (Bohlin DSR II, Malvern Panalytical Ltd., Malvern, UK) experiment at a frequency of 10 rad/s in line with the ASTM D7175 standard at moderate and high temperatures to determine the viscous and elastic properties.

The DSR testing was performed on specimens with a thickness of 1.0 mm and a diameter of 25.0 mm at high service temperatures ranging from 52 to 76 °C. The rutting characteristics (G*/sinδ) and the performance of the bitumen binder were investigated. Similarly, the pressure aging vessel-aged bitumen were assessed at 18, 23, 25, and 29 °C under shear loading to evaluate performance at medium service temperatures and fatigue characteristics (G*.sinδ). The optimum G*.sinδ requirement for long-term, PAV-aged PMBs specimens is 5000 kPa. For fatigue cracking resistance (FCR), high δ and low G* values are required, however for permanent deforming resistance (PDR), low δ and high G* values are desirable [46].

##### Rotational Viscosity Test

Rotational viscometer (DV-III, Brookfield, Harlow, UK) is also used in this research to investigate the flowing parameters of PC and PTFE-modified bitumen at high-temperature levels as a measurement of flowability, mixability, and deformability according to standard ASTM D4402 methods. Even though the RV test can be conducted at varying temperatures, the Asphalt Institute recommends measuring binder viscosity at 275 °F (135 °C). It was proposed that temperature limits dependent on viscosity restraints of 280 ± 30 cP and 170 ± 20 cP be used to assess compressive and mixing temperatures, correspondingly [47].

##### Bending Beam Rheometer Test

At low air temperatures, the BBR test (BBR 2, ATS, Butler, PA, USA) was used to determine the thermal splitting strength and rigidity of PMBs. BBR experiment was used to evaluate the creep stiffness as a function of time, which is evidence of the stiffness of the binder and to measure the change in the hardness of the binder known as m-value. For creep stiffness, superpave specified a maximum of 300 MPa and a minimum of 0.300 m-value [48]. In accordance with ASTM D6648, the PAV-aged test samples were introduced to this analysis to explore the effects of PC and PTFE-modified bitumen on the lower temperature efficiency parameters of the binders.

### 2.3. Analytical Characterization

#### 2.3.1. Scanning Electron Microscopy

Scanning electron microscopy is a microscopic method that uses a precisely focused beam of electron to show numerous aspects of a specimen related to surface features (size, shape, and uniformity) of the PMBs examined particles. SEM seems to be the most useful tool for determining the compatibility of bitumen additives, determining the best polymer quantity, and analyzing the damage produced by the aging cycle. In this study, the microstructure of PMBs specimens was examined using JSM 5910 JEOL Ltd., Tokyo, Japan, at a 20 kV electron acceleration voltage with a magnification of 100 µm. The traditional bitumen-rich layer displays a dark or black color in SEM images. In contrast, the modifiers-rich PC and PTFE layers exhibit white colored additions [49].

#### 2.3.2. FTIR Spectroscopy Analysis

In this research, atomic structures of PCMB, PTFEMB, and PCMB-PTFEMB specimens were investigated using the FTIR analytical method with the help of a spectrometer (Nexus 870, Thermo Nicolet corps, Waltham, MA, USA). To prepare solution of CS_2_/Bitumen, the neat bitumen and modified specimens were firstly dipped in (CS_2_) at a dosage of 5% by weight, and then with the help of FTIR spectrometer, FTIR Spectra were recorded in a wavenumber range of 4000–450 cm^−1^.

#### 2.3.3. XRD Analysis

For characterize of neat and modified bitumen, continuous X-ray diffraction patterns of PMBs specimens were carried out at an intermediate temperature with scanning range from 5.0 to 80° 2θ at step size of 0.02° by using Schimadzu X-ray Diffraction-6000 device (Shimadzu Corporation, Tokyo, Japan), with the operational condition of Cu-K closed tube (k = 0.154057 nm) executed at 40.0 kV and 30.0 mA.

## 3. Results and Discussion

### 3.1. Physical and Rheological Properties Results

By utilizing different concentrations (0%, 2.5%, and 5% by weight) of PC and PTFE as modifying agents, the physical and rheological characterization of traditional and modified bituminous binder’s specimens before and after aging were determined.

**Figure 1:**
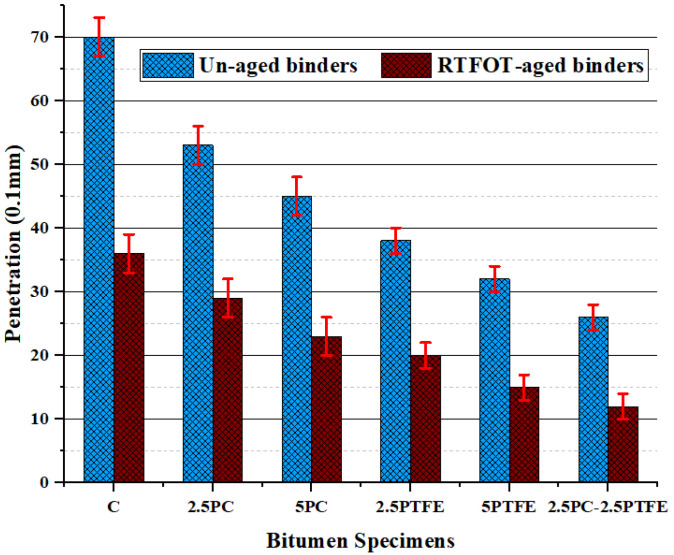
Penetration test values of binder before and after aging.

**Figure 2:**
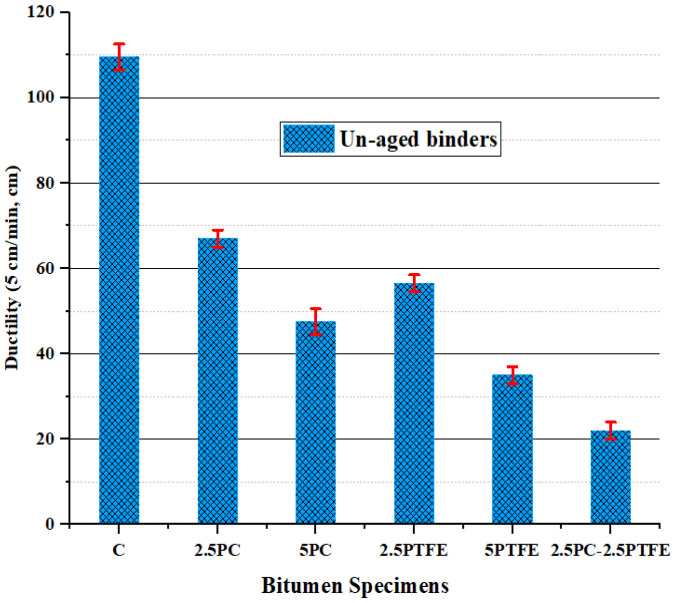
Ductility test values of un-aged binders.

**Figure 3:**
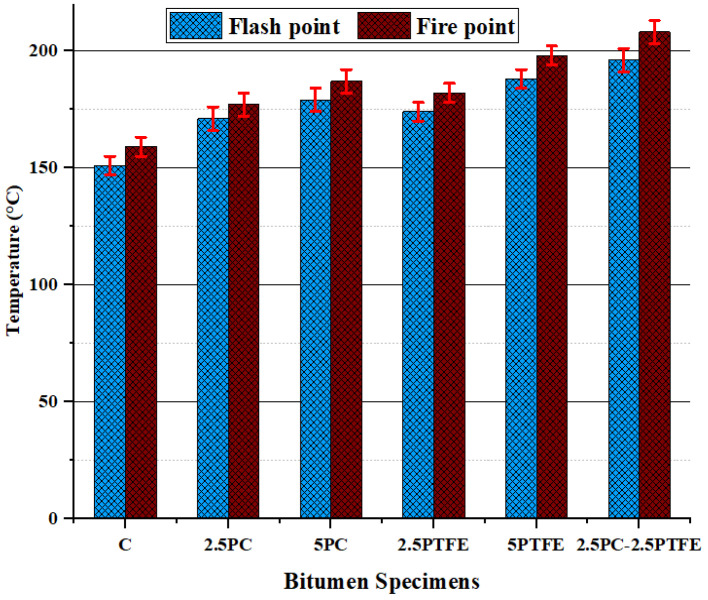
Flash and fire point temperature values of binders.

**Figure 4:**
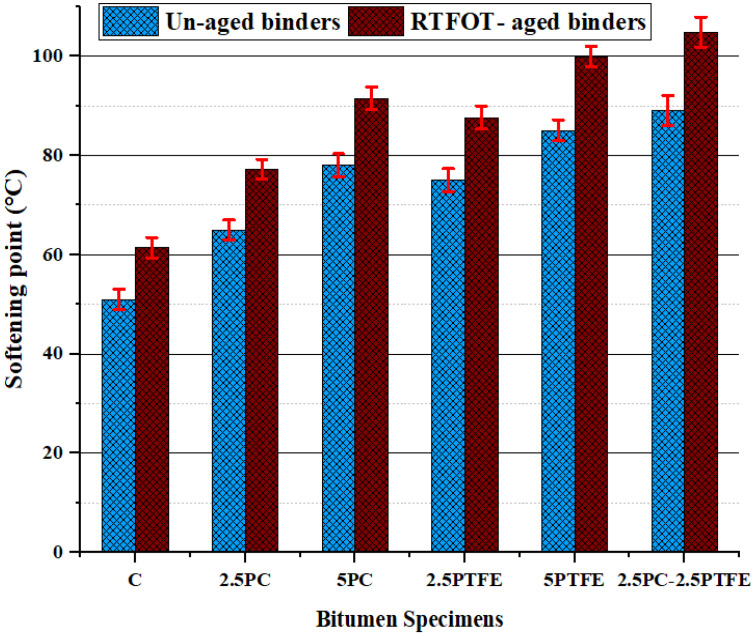
The SPT value of binders before and after aging.

When 2.5% and 5% of each synthetic thermoplastic polymer, PC and PTFE, were added to the neat bitumen separately and 2.5% mixed by weight as modifiers to the pure bitumen, Figure 1 and Figure 2 show that its penetration values and ductility for PCMB was decreased by 23.2%, 33.3%, 38.8%, and 56.6%, respectively. Additionally, test results presented in Figure 1 and Figure 2 depict that as we increase the PTFE content in the blend, it also decreases the penetration values by 44.9%, 56.5%, and ductility values by 48.4%, 68%, respectively, and for PTFEMB samples, ductility decreased by 79.9% and penetration decreased by 62.3%. Modifying neat grades bitumen by PC and PTFE, on the other hand, increased the fire point and softening point temperatures of PMBs from 159 °C to 208 °C (i.e., 30.8% increase) and 51 °C to 90 °C (i.e., 76.5% increase) correspondingly, as shown in Figure 3 and Figure 4.

**Figure 5:**
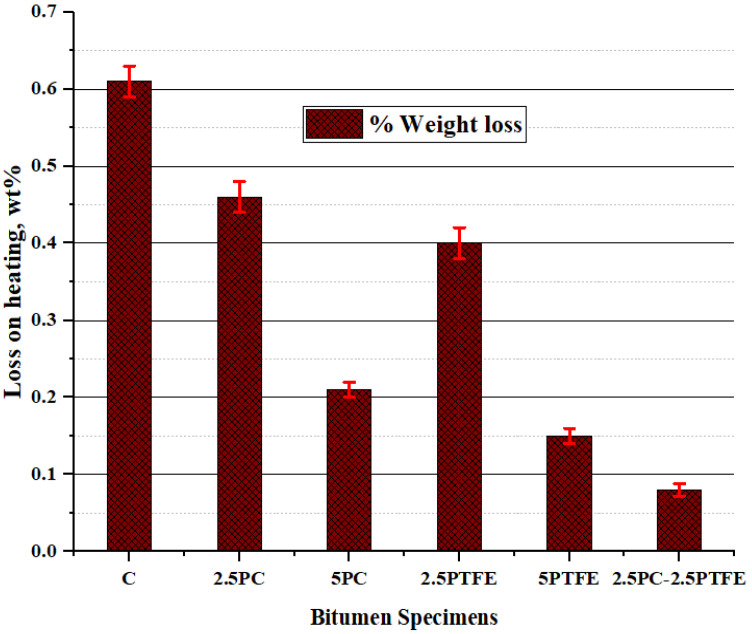
The weight loss on heating of the binders after RTFOT.

The rise in SPT value, flash, and fire point temperature, and decline in penetration and ductility values, show a substantial reduction in temperature sensitivity and a substantial improvement in the stiffness characteristics of the bituminous mix, as assessed by the neat bitumen test results. Using synthesized polymers, polycarbonate, and polytetrafluoroethylene as a modifying agent in neat bitumen decreased the penetration value of a neat grade 70 bitumen binder, according to the findings of the experiments. Furthermore, the higher the concentration of modifiers, the stiffer the asphalt binder will be. Therefore, it is reasonable to state that asphalt binders with a soft consistency used in frigid zones could be used in warmer zones by varying the concentrations of PC and PTFE. Figure 5 illustrates that PCMB, PTFEMB, and PCMB-PTFEMB binders lose considerably less weight than base binders. This loss is also lower than 1%, the upper limit value. Besides this, increasing the percentage of modifiers reduces mass loss, which was found to be 65.60%, 75.40%, and 86.80% accordingly, due to the varying percentages of PC and PTFE by weight [45].

Adding PC and PTFE as modifiers makes the asphalt binders less aged or harder. The chemical reaction between the neat bitumen and modifiers increases the modified bitumen’s resistance to temperature and oxidation.

**Figure 6:**
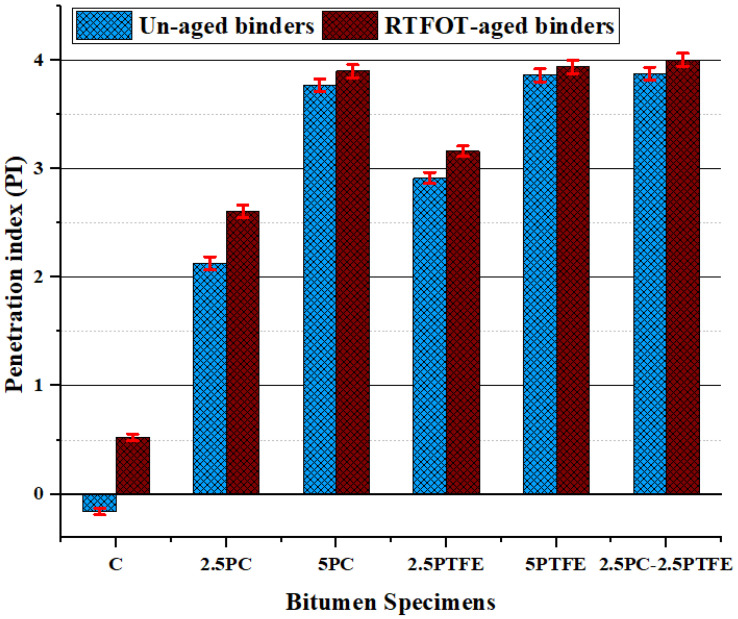
The penetration index values of binders before and after aging.

When the influence of PC and PTFE on the penetration index of the bitumen binders is studied, it is shown that PMBs have higher PI values than neat bitumens grade, with an increase of 1.83%, 6.2%, and 2.5% based on the additive amounts, as presented in Figure 6. Therefore, this implies that PC and PTFE-modified bitumen with greater PI values can resist fracturing at low temperatures and provide more resistance against permanent deformations and thermal cracking at elevated temperatures [50].

### 3.2. Dynamic Shear Rheometer Test

**Figure 7:**
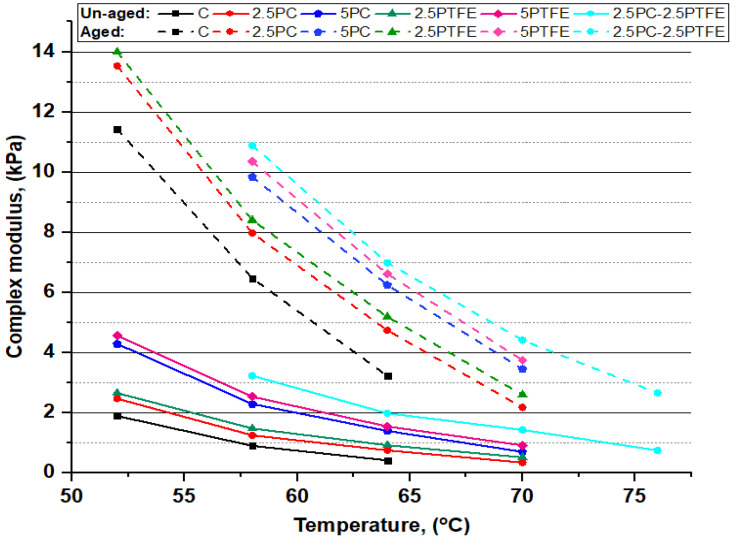
Relationship between complex modulus (G*) and temperature before and after aging.

**Figure 8:**
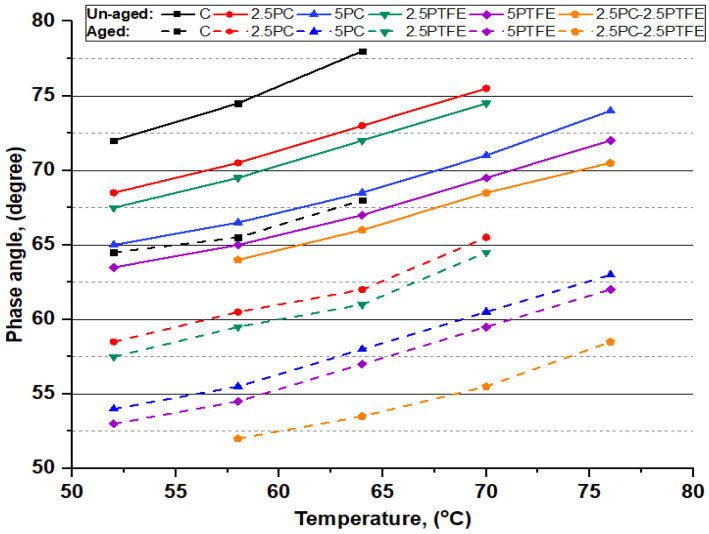
The phase angle (δ) and temperature relationship of binders before and after aging.

**Figure 9:**
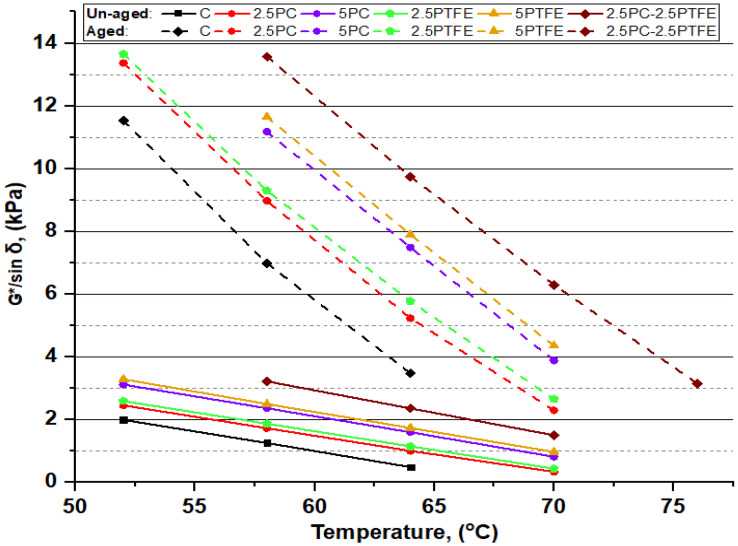
G*/sinδ and temperature relationship of binders before and after aging.

**Figure 10:**
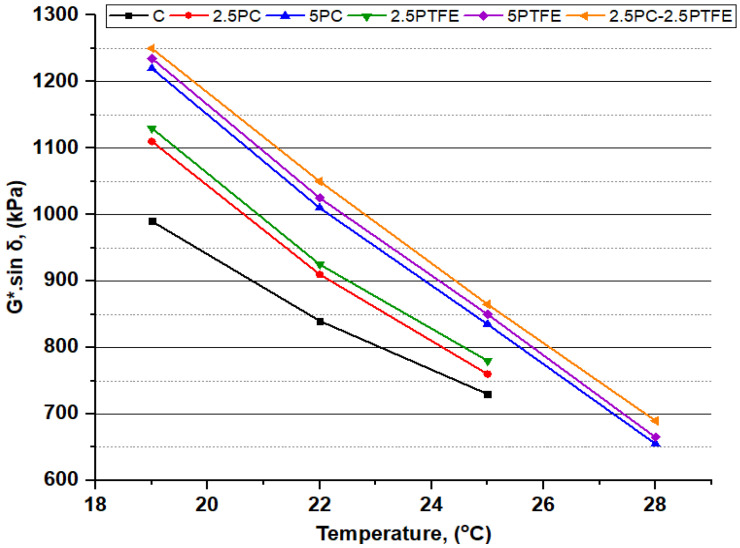
G*.sinδ and temperature relationship of all the PAV-aged binders.

The dynamic shear rheometer test was carried out to investigate the rheological properties (G*) of neat grades of bitumen and PCMB, PTFEMB, and PCMB-PTFEMB samples at intermediate and high service temperatures. For high temperatures, rutting resistance factor (G*.sinδ) values were used to evaluate bitumen specimens performance. In contrast, fatigue resistance parameters (G*.sinδ) of the bitumen binder were investigated for moderate temperatures [51]. DSR testing results of neat and PMBs samples at various temperatures presented in Figure 7, Figure 8, Figure 9 and Figure 10 depict the relationship between temperature and G*, δ, G*/sinδ, and G*.sinδ accordingly.

Figure 7 shows that as the PC and PTFE content increases in the blend, the complex modulus (G*) values in both aged and un-aged bitumen specimens have dramatically increased, depending on the percentage of modifiers, but decreased with the increasing temperature. However, the results presented in Figure 8 show that phase angle (δ) values for all the bitumen specimens decreased with increasing the percentages of PC and PTFE in neat bitumen but increased with temperature, indicating that PC and PTFE-modified bitumen have a higher elasticity. Moreover, a more minor phase angle (δ) and higher (G*) value indicates that polymer-modified bitumen has significantly better resistance to permanent deformations.

The rutting parameter (G*.sinδ) values of PC and PTFE-modified bitumen were higher than the plain bitumen values at the same temperature, as shown in Figure 9. Furthermore, Figure 10 also illustrates that for all bitumen specimens, the values of the fatigue resistance parameter (G*.sinδ) stayed below the 5000 kPa limit value, indicating that the fatigue cracking resistance of PMBs is satisfactory at intermediate temperatures [52]. From these results, it can be said that asphalt pavement may not be subjected to binder-induced fatigue cracks due to repeated loads.

The dynamic shear rheometer tests determined that by adding the bitumen modifiers, PC and PTFE, bitumen stiffness increased. At the same time, temperature sensitivity decreased, implying that modified bitumen had greater permanent deformation resistance at elevated temperature ranges.

### 3.3. Rotational Viscosity Test

**Figure 11:**
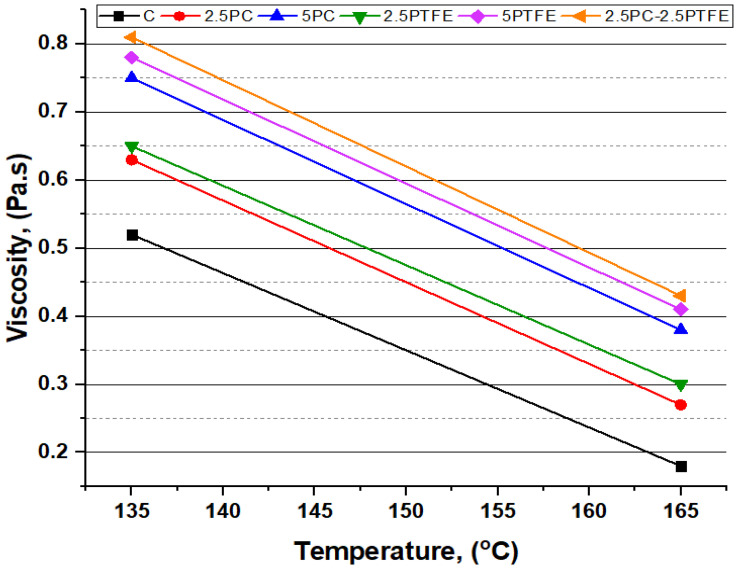
Temperature–viscosity relationship of binders.

The outcomes of the RV test, shown in Figure 11, reveal that when compared to pure bitumen, the modified bitumen exhibits higher viscosity values of all bitumen samples alongside an increase in polycarbonate and polytetrafluoroethylene content in the blend, indicating that the binders’ flow ensures pumping safety. In comparison to pure viscosity grade 70 bitumen, RV test results for PCMB, PTFEMB, and PCMB-PTFEMB increased by 44%, 50%, and 55.75% at 135 °C, and 111.10%, 127.8%, and 138.88% at 165 °C, respectively, as bitumen-modifier content is increased [53].

Furthermore, with increasing rotational viscosity, the mixing and compaction temperatures of PCMB, PTFEMB, and PCMB-PTFEMB-modified bitumen increased by 3.33%, 4.67%, and 5.33% accordingly, which implies that the addition of modifiers, PC and PTFE, in neat grades of bitumen increased the viscosity and stifness of bitumen blend significantly, corresponding to percentages of modifiers.

### 3.4. Bending Beam Rheometer Test

**Figure 12:**
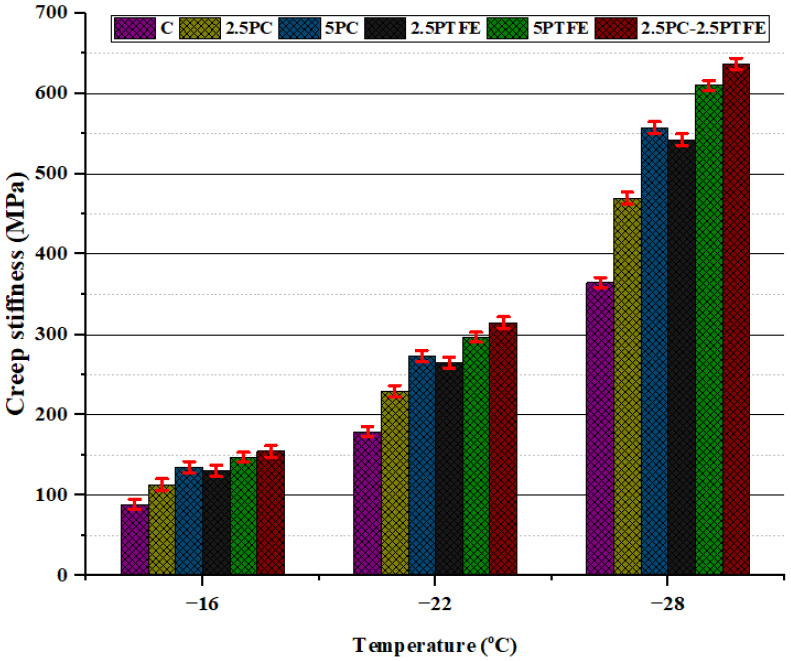
Effects of PC and PTFE on the creep stiffness of PG 70.

**Figure 13:**
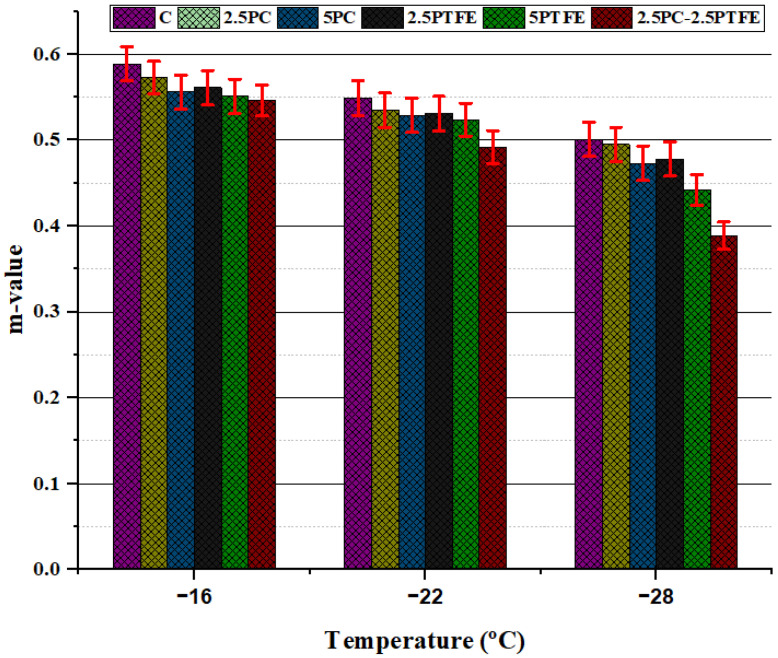
Effects of PC and PTFE on the m-value of PG 70.

The creep stiffness modulus (S) and greater creep ratio (m-value) values were obtained by utilizing the BBR tester. BBR results for neat and modified bitumens specimens presented in Figure 12 and Figure 13 demonstrate the relationship of creep stiffness modulus (S) and creep ratio (m-value) at various temperature ranges, respectively.

Creep stiffness of PC and PTFE-modified bitumen increased at low temperatures due to a rise in the percentage content of modifiers in the blend compared to neat bitumen, as shown in Figure 12, indicating that bitumen becomes more rigid as a result of additives in blends. On the other hand, as shown in Figure 13, m-values declined as the additive rate increased [48]. According to these findings, it was determined that cracking may occur in modified bitumen specimens at low temperatures.

### 3.5. Scanning Electron Microscopy

**Figure 14:**
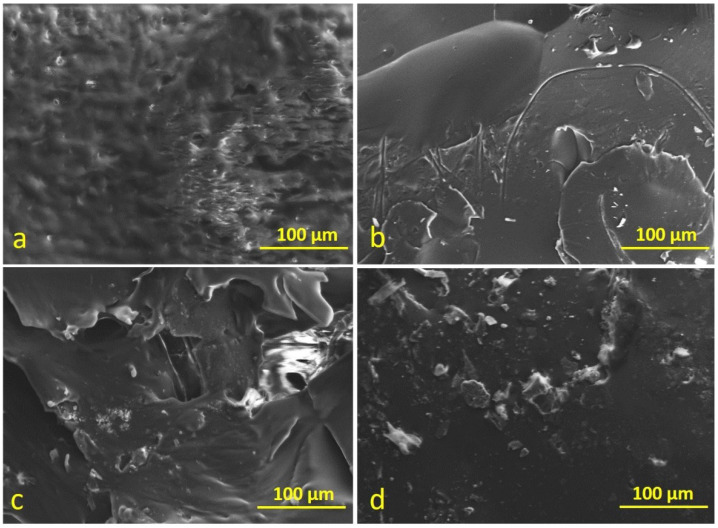
SEM images of bitumen specimens before aging: (**a**) C, (**b**) 5PC, (**c**) 5PTFE, (**d**) 2.5PC-2.5PTFE.

**Figure 15:**
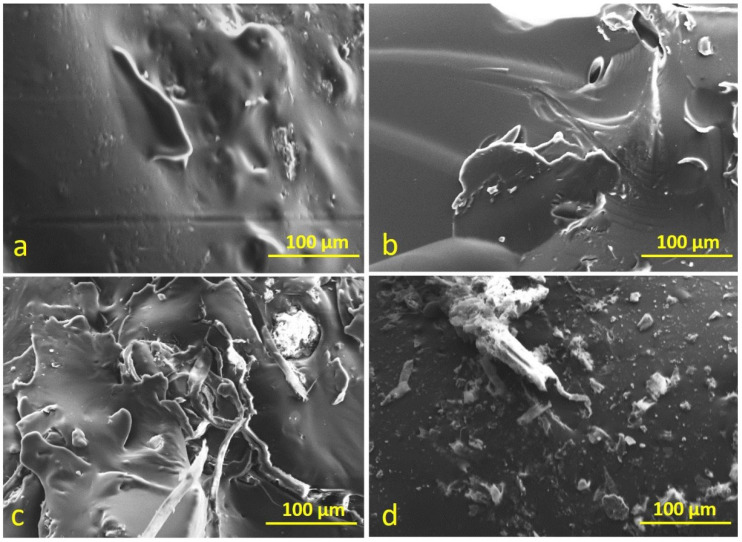
SEM images of bitumen specimens after RTFO- aging: (**a**) C, (**b**) 5PC, (**c**) 5PTFE, (**d**) 2.5PC-2.5PTFE.

SEM images of neat and PMBs showed in Figure 14 and Figure 15 were obtained using scanning electron microscopy, which allows for the monitoring of molecular structure and homogeneity of the blend, to assess the dispersion condition of PC and PTFE in neat bitumen, to explore the microstructure of modified bitumen, and to classify the structure of the continuous and undefined cycle [54,55].

Figure 14a–d illustrate that the structure of neat bitumen obtained from SEM is a single-phase homogeneous. Images of PC and PTFE-modified bitumen show irregular particles pattern and regular bulges around the particles, respectively. In contrast, images of mixes (PC and PTFE) modified bitumen specimen displays regular concave circles around the particle. SEM images of RTFOT-aged modified bitumen presented in Figure 15a–d shows that neat and modified bitumen changed into an amorphous form as the regular patterned structures can be seen in SEM images. SEM images revealed structural changes on the surface of PC and PTFE-modified bitumen when compared to the neat sample, indicating the possibility of a chemical reaction between neat bitumen and synthetic polymers or the loss of some minor molecular groups from the structure of bitumen due to aging.

### 3.6. Fourier Transform Infrared Spectroscopy

**Figure 16:**
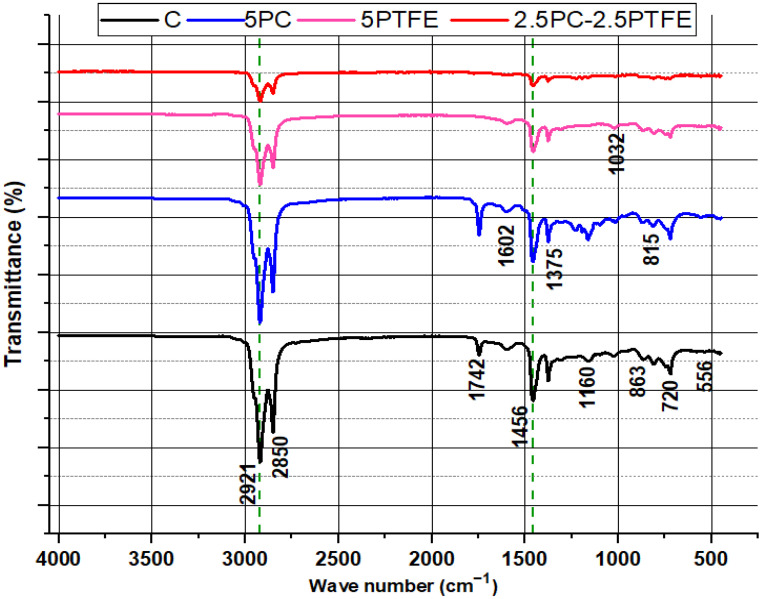
FTIR spectra of un-aged bitumen samples.

**Figure 17:**
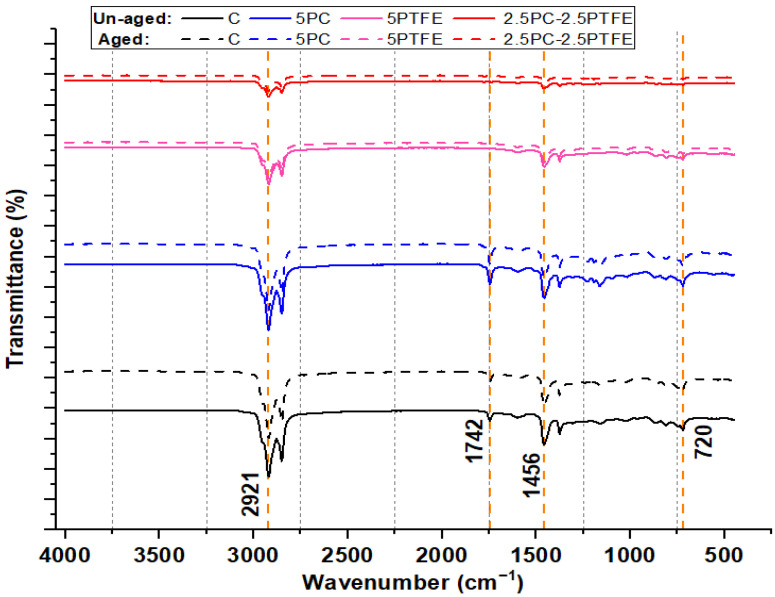
FTIR spectroscopy spectra of RTFOT aged specimens relative to bitumen specimens before aging process.

Figure 16 and Figure 17 show the FTIR spectra of neat and PMBs-modified specimens (PCMB, PTFEMB, and PCMB-PTFEMB) before and after aging, and characteristic peaks can be used to identify molecular structures. The FTIR spectra of the neat and PMBs, as shown in Figure 16, reveal twelve distinct spectral bands. The transmittance of wide range of molecular structures is represented by spectral bands at unique wavenumbers. The strongest absorption peaks at 2921 cm^−1^ and 2850 cm^−1^ appear in the spectra of all the binders. The asymmetric stretching vibration of C-H causes peaks at 2921 cm^−1^, while the symmetrical stretching vibration of C-H causes peaks at 2850 cm^−1^. Furthermore, Figure 16 shows the FTIR spectra of the base and modified-bitumen, which reveals that there was no water retention in the samples because the O-H groups have no peak at wavenumbers greater than 3150 cm^−1^.

The aromatic structure is characterized by the breathing vibration of asymmetrically substituted benzene (spectral bands at around 1602 cm^−1^). In contrast, the branched aliphatic structure is characterized by the bending vibration of methyl C-H (spectral bands at around 1375 cm^−1^ and 1456 cm^−1^). The bending vibration absorption spectrum of the aromatic tape in the original asphalt is represented by peaks at 815 cm^−1^. Peaks 863, 720, and 556 cm^−1^ also show aromatic para, meta, and ortho functional groups, respectively. Stretching vibration of sulphoxide (S=O) produces a smaller peak at about 1032 cm^−1^ in all FTIR spectra [56], and spreading vibrations of sulphoxide bonds (S–O) were found at 1160 cm^−1^. The peak at 863 cm^−1^ remained consistent throughout the reaction (at both 20 and 65 °C) and appeared due to crystalline matter (fayalite, diopside, clinoferrite). The bending vibration of Si-O-Si and Si-O-Al produces peak intensities in the 400 cm^−1^ –600 cm^−1^ range [56,57]. After RTFOT aging, the peak positions and intensities of varying peaks in the FTIR spectra of neat and modified-bitumen is presented in Figure 17. It was observed that peak positions of the curves were quite similar to un-aged specimens, but peak values of the FTIR spectra at wavenumbers ranging from 1700 to 400 cm^−1^ were slightly decreased due to aging and the presence of PC and PTFE polymers, indicating that bitumen chemical composition has changed, which leads to an increase in SPT values and a decrease in penetration values.

### 3.7. X-ray Diffraction Analysis

**Figure 18:**
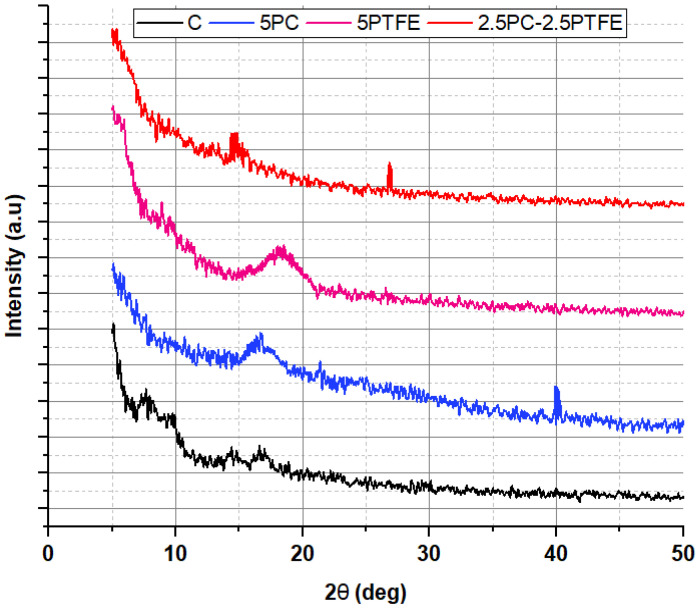
X-ray diffraction pattern binders before aging.

**Figure 19:**
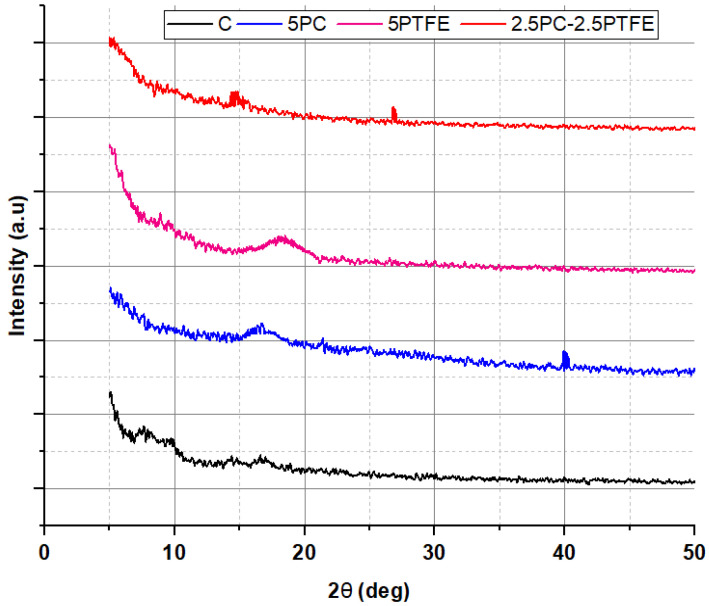
X-ray diffraction patterns of binders after aging.

Figure 18 and Figure 19 exhibit XRD patterns for un-aged and RTFOT-aged specimens prepared from neat and modified bitumen, respectively. The XRD spectra of modified and neat bitumen, as shown in Figure 18. The broad XRD band, where 2-theta is between 15.5° and 21.5°, clearly shows the amorphous structure. The altitude of the peak reveals that the structure contains crystal shadows.

Two peaks were identified for PC-modified bitumen XRD spectra, as shown in Figure 18—one broad peak at (17.2°) and another sharp peak at a stage where the 2θ value is about 40°. For the PTFEMB, only one peak was identified at a point where the 2-theta value is 18.1°, which agrees with the findings published for thin PTFE coatings with thicknesses of 94, 142 nm [58]. This implies that PTFE interacts with the bitumen’s main macro-molecular groups and is distributed evenly throughout the mixture. The crystalline structure of RTFOT-aged bitumen decreased with the utilization of modifiers, PC and PTFE, into neat grades of bitumen, as shown in Figure 19. In other words, the bitumen blend was dominated by the amorphous structure formed by the reaction of bitumen and additive.

**Figure 20:**
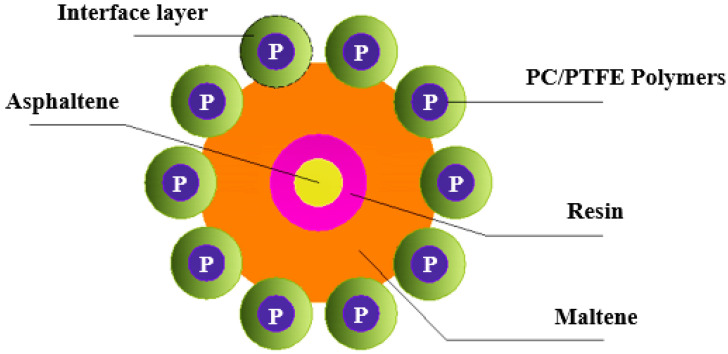
A plausible interaction mechanism between bitumen and PC/PTFE polymers.

The plausible interaction mechanism between polymeric additions (Polycarbonate and Polytetrafluoroethylene) and bitumen is represented in the schematic, as shown in Figure 20. Swelling is primarily a process of interdiffusion between light components of asphalt and polymer molecules at the asphalt-aggregate interface, which is caused by the high-viscosity modifier adsorbing light components of asphalt. A polymer-centered interface layer will result from this.

## 4. Conclusions

Pavement engineers can better understand bitumen mechanical behavior at a relatively larger scale if they have a comprehensive knowledge of its viscoelastic, physical, and micro-mechanical properties. The influence of polycarbonate PC and PTFE, a manufactured thermoplastic polymer, on the engineering parameters of PG 70 bitumen were comprehensively investigated in this research, with the main findings summarized as follows.

1. Flash and fire point and softening point temperatures substantially increased as the PC. PTFE concentration in the bitumen mix increased slightly. At the same time, ductility and penetration values dramatically decreased, indicating that the binder has become considerably harder by modification and is much more appropriate for high service temperatures. The chemical reaction between neat bitumen and modifiers, PC and PTFE, is the cause of the rheological behavioral modification.
2. The RTFOT findings illustrate that increasing the percentages of PC and PTFE in the mixture reduces bitumen aging, indicating that PMBs dissipate less mass during the blending and compressing stage. Furthermore, this explains that the chemical change in mixes improves the PMBs resistance to temperature and oxidation.
3. The complex modulus (G*) of un-aged and aged modified-bitumen increased with increasing modifiers percentages, whereas phase angle (δ) values declined significantly, signifying that the modified binder was stiffer (higher G* values) and comparatively more elastic solid (lower δ values). SUPERPAVE suggested RTFOT-aged, and PAV-aged asphalt binder values as the rutting parameter (G*/sinδ) and fatigue parameter (G*.sinδ) shall be 2.2 kPa and 5000 kPa, respectively. The (G*.sinδ) and (G*/sinδ) values obtained are from 655 to 1255 kPa and 2.289 to 13.653 kPa respectively. According to the results, it is possible to say that PC and PTFE-modified bitumen would be more suitable to use in hot climate regions because binders become stiff enough to resist rutting, fatigue failures, and permanent deformations.
4. The combination of modifiers, PC and PTFE, with neat bitumen increased the neat bitumen viscosity for PCMB, PTFEMB, and PCMB-PTFEMB by 44%, 50%, and 55.75% at 135°C, and 111.10%, 127.80%, and 138.88% at 165 °C accordingly. The findings reveal that PC and PTFE decreased the flow parameters and increased the stiffness characteristics of neat grades of bitumen.
5. BBR test results revealed that with the application of the load, the hardness of the PC and PTFE-modified bitumen exposed to low-temperature ranges and the rate of their hardening speed significantly increased. BBR results show that as modifiers’ content increases, the base binder’s creep stiffness values increased by 74.8%, 75.8%, and 74.5% at −16 °C, −22 °C, and −28 °C, respectively, which indicates that bitumen becomes more rigid and stiff by modification.
6. From the result of chemical reaction in the bitumen mix, SEM, X-ray Diffraction, and FTIR studies showed that modifiers, PC and PTFE, interacted with the primary macro-molecular groups of the neat bitumen, eventually resulting in a homogenous single-phase structure.

## Figures and Tables

**Table 1:** The properties of PG 70 neat bituminous material.

Sr. No	Physical Properties	Temperature Value (°C)	ASTM Methods	Values
1	Penetration grade (100 g, 5 s, 0.1 mm)	25	D5	70
2	Ductility value test (5 cm/min, cm)	25	D113	109.5
3	SPT value, °C	-	D36	51
4	FPT1 value, °C	-	D92	151
5	FPT2, °C	-	D92	159
6	Specific Gravity (g/cm^3^)	25	D70	1.035
7	Absolute viscosity (Pa.s)	135	D4402	0.52
8	Penetration index	-	-	−0.16

**Table 2:** Physical, Rheological, and Mechanical properties of PC.

Sr. No	Properties/Units	Values
1	Specific Gravity, g/cm^3^	1.20
2	Elongation at break, %	111
4	Ultimate Tensile Strength, MPa	65.50
5	Elastic Modulus, GPa	2.3786
6	Compressive Strength, MPa	85.0

**Table 3:** Physical, Rheological, and Mechanical properties of PTFE.

Sr. No	Properties/Units	Values
1	Specific Gravity, g/cm^3^	2.164
2	Elongation at break, %	451
3	Shear Strength, MPa	4.98
4	Ultimate Tensile Strength, MPa	31.0
5	Elastic Modulus, MPa	30.9
6	Compressive Strength, MPa	23.5

## Data Availability

The data presented in this study are available on request from the corresponding author.

## References

[1] Jiang W., Sha A., Xiao J., Li Y., Huang Y. (2015). Experimental study on filtration effect and mechanism of pavement runoff in permeable asphalt pavement. Constr. Build. Mater..

[2] Xu G., Wang H. (2017). Molecular dynamics study of oxidative aging effect on asphalt binder properties. Fuel.

[3] Zhang H., Chen Z., Li L., Zhu C. (2017). Evaluation of aging behaviors of asphalt with different thermochromic powders. Constr. Build. Mater..

[4] Chen J.-S., Liao M.-C., Shiah M.-S. (2002). Asphalt modified by styrene-butadiene-styrene triblock copolymer: Morphology and model. J. Mater. Civ. Eng..

[5] Rubio M.C., Martínez G., Baena L., Moreno F. (2012). Warm mix asphalt: An overview. J. Clean. Prod..

[6] Durrieu F., Farcas F., Mouillet V. (2007). The influence of UV aging of a styrene/butadiene/styrene modified bitumen: Comparison between laboratory and on site aging. Fuel.

[7] Wu S., Pang L., Liu G., Zhu J. (2010). Laboratory study on ultraviolet radiation aging of bitumen. J. Mater. Civ. Eng..

[8] Xiao F., Newton D., Putman B., Punith V., Amirkhanian S.N. (2013). A long-term ultraviolet aging procedure on foamed WMA mixtures. Mater. Struct..

[9] Mouillet V., Farcas F., Besson S. (2008). Ageing by UV radiation of an elastomer modified bitumen. Fuel.

[10] Zeng W., Wu S., Wen J., Chen Z. (2015). The temperature effects in aging index of asphalt during UV aging process. Constr. Build. Mater..

[11] Xiao F., Chen M., Wu S., Amirkhanian S.N. (2013). A Long-Term Ultraviolet Aging Effect on Rheology of WMA Binders. Int. J. Pavement Res. Technol..

[12] Xue Y., Ge Z., Li F., Su S., Li B. (2017). Modified asphalt properties by blending petroleum asphalt and coal tar pitch. Fuel.

[13] Robinson H. (2005). Polymers in Asphalt.

[14] Apostolidis P., Liu X., Kasbergen C., Scarpas A.T. (2017). Synthesis of asphalt binder aging and the state of the art of antiaging technologies. Transp. Res. Rec..

[15] Ling M., Luo X., Chen Y., Gu F., Lytton R.L. (2020). Mechanistic-empirical models for top-down cracking initiation of asphalt pavements. Int. J. Pavement Eng..

[16] Ling M., Chen Y., Hu S., Luo X., Lytton R.L. (2019). Enhanced model for thermally induced transverse cracking of asphalt pavements. Constr. Build. Mater..

[17] Li X., Ouyang C., Yuan Y., Gao Q., Zheng K., Yan J. (2015). Evaluation of ethylene–acrylic acid copolymer (EAA)-modified asphalt: Fundamental investigations on mechanical and rheological properties. Constr. Build. Mater..

[18] Ameri M., Mansourian A., Ashani S.S., Yadollahi G. (2011). Technical study on the Iranian Gilsonite as an additive for modification of asphalt binders used in pavement construction. Constr. Build. Mater..

[19] Airey G.D. (2003). Rheological properties of styrene butadiene styrene polymer modified road bitumens☆. Fuel.

[20] Airey G.D., Mohammed M.H., Fichter C. (2008). Rheological characteristics of synthetic road binders. Fuel.

[21] Yildirim Y. (2007). Polymer modified asphalt binders. Constr. Build. Mater..

[22] Abtahi S.M., Sheikhzadeh M., Hejazi S.M. (2010). Fiber-reinforced asphalt-concrete—A review. Constr. Build. Mater..

[23] Bazlamit S.M., Reza F. (2005). Changes in asphalt pavement friction components and adjustment of skid number for temperature. J. Transp. Eng..

[24] Fischer H.R., Cernescu A. (2015). Relation of chemical composition to asphalt microstructure–Details and properties of micro-structures in bitumen as seen by thermal and friction force microscopy and by scanning near-filed optical microscopy. Fuel.

[25] Al-Rub R.K.A., Darabi M.K., Little D.N., Masad E.A. (2010). A micro-damage healing model that improves prediction of fatigue life in asphalt mixes. Int. J. Eng. Sci..

[26] Appiah J.K., Berko-Boateng V.N., Tagbor T.A. (2017). Use of waste plastic materials for road construction in Ghana. Case Stud. Constr. Mater..

[27] Hou X., Lv S., Chen Z., Xiao F. (2018). Applications of Fourier transform infrared spectroscopy technologies on asphalt materials. Measurement.

[28] Jin J., Tan Y., Liu R., Lin F., Wu Y., Qian G., Wei H., Zheng J. (2018). Structure characteristics of organic bentonite and the effects on rheological and aging properties of asphalt. Powder Technol..

[29] Zhang H., Chen Z., Xu G., Shi C. (2018). Evaluation of aging behaviors of asphalt binders through different rheological indices. Fuel.

[30] Zhu J., Birgisson B., Kringos N. (2014). Polymer modification of bitumen: Advances and challenges. Eur. Polym. J..

[31] Downturn A.M. (2012). Global Demand for Polycarbonate Growing Again, Says IHS Chemical Report.

[32] Jerabek M., Major Z., Lang R.W. (2010). Strain determination of polymeric materials using digital image correlation. Polym. Test..

[33] Van Der Walt I., Bruinsma O. (2006). Depolymerization of clean unfilled PTFE waste in a continuous process. J. Appl. Polym. Sci..

[34] Gama D.A., Júnior J.M.R., de Melo T.J.A., Rodrigues J.K.G. (2016). Rheological studies of asphalt modified with elastomeric polymer. Constr. Build. Mater..

[35] Jasso M., Hampl R., Vacin O., Bakos D., Stastna J., Zanzotto L. (2015). Rheology of conventional asphalt modified with SBS, Elvaloy and polyphosphoric acid. Fuel Process. Technol..

[36] Polacco G., Filippi S., Merusi F., Stastna G. (2015). A review of the fundamentals of polymer-modified asphalts: Asphalt/polymer interactions and principles of compatibility. Adv. Colloid Interface Sci..

[37] De Sá M.d.F.A., Lins V.d.F.C., Pasa V.M.D., Leite L.F.M. (2013). Weathering aging of modified asphalt binders. Fuel Process. Technol..

[38] Ge D., Yan K., You L., Wang Z. (2017). Modification mechanism of asphalt modified with Sasobit and Polyphosphoric acid (PPA). Constr. Build. Mater..

[39] Gama D.A., Yan Y., Rodrigues J.K.G., Roque R. (2018). Optimizing the use of reactive terpolymer, polyphosphoric acid and high-density polyethylene to achieve asphalt binders with superior performance. Constr. Build. Mater..

[40] Seyfullah K. (2010). Investigation of penetration and penetration index in bitumen modified with SBS and reactive terpolymer. Sigma.

[41] Topal A. (2010). Evaluation of the properties and microstructure of plastomeric polymer modified bitumens. Fuel Process. Technol..

[42] Keyf S. (2015). The modification of bitumen with reactive ethylene terpolymer, styrene butadiene styrene and variable amounts of ethylene vinyl acetate. Res. Chem. Intermed..

[43] Irfan M., Saeed M., Ahmed S., Ali Y. (2017). Performance evaluation of elvaloy as a fuel-resistant polymer in asphaltic concrete airfield pavements. J. Mater. Civ. Eng..

[44] Porto M., Caputo P., Loise V., Eskandarsefat S., Teltayev B., Oliviero Rossi C. (2019). Bitumen and bitumen modification: A review on latest advances. Appl. Sci..

[45] Geckil T., Ahmedzade P., Alatas T. (2018). Effect of carbon black on the high and low temperature properties of bitumen. Int. J. Civ. Eng..

[46] Zaniewski J.P., Pumphrey M.E. (2004). Evaluation of performance graded asphalt binder equipment and testing protocol. Asph. Technol. Program.

[47] West R.C., Watson D.E., Turner P.A., Casola J.R. (2010). Mixing and Compaction Temperatures of Asphalt Binders in Hot-Mix Asphalt.

[48] Nicholls C. (2007). Analysis of available data for validation of bitumen tests: Report on phase 1 of the BiTVAL project. Advanced Course on Bitumen Technology.

[49] Mazumder M., Ahmed R., Ali A.W., Lee S.-J. (2018). SEM and ESEM techniques used for analysis of asphalt binder and mixture: A state of the art review. Constr. Build. Mater..

[50] Taherkhani H., Afroozi S. (2016). The properties of nanosilica-modified asphalt cement. Pet. Sci. Technol..

[51] Celauro B., Celauro C., Presti D.L., Bevilacqua A. (2012). Definition of a laboratory optimization protocol for road bitumen improved with recycled tire rubber. Constr. Build. Mater..

[52] Baklokk L., Skoglund R., Kalman B., Peltonene P. (2002). Superpave Test Methods for Asphalt–Procedure for DSR Testing.

[53] Ahmedzade P. (2013). The investigation and comparison effects of SBS and SBS with new reactive terpolymer on the rheological properties of bitumen. Constr. Build. Mater..

[54] Vo H.V., Park D.-W. (2017). Application of conductive materials to asphalt pavement. Adv. Mater. Sci. Eng..

[55] Lamontagne J., Dumas P., Mouillet V., Kister J. (2001). Comparison by Fourier transform infrared (FTIR) spectroscopy of different ageing techniques: Application to road bitumens. Fuel.

[56] Zhang H., Yu J., Wu S. (2012). Effect of montmorillonite organic modification on ultraviolet aging properties of SBS modified bitumen. Constr. Build. Mater..

[57] Hrachová J., Komadel P., Fajnor V.Š. (2007). The effect of mechanical treatment on the structure of montmorillonite. Mater. Lett..

[58] Wang J., Kim H., Shi F.G., Zhao B., Nieh T. (2000). Thickness dependence of morphology and mechanical properties of on-wafer low-k PTFE dielectric films. Thin Solid Film..

